# Association Between Internet Addiction and Comorbid Anxiety and Depression in Chinese Children and Adolescents: A Latent Profile Analysis and Network Analysis

**DOI:** 10.3390/healthcare14070862

**Published:** 2026-03-27

**Authors:** Tingting Xiao, Yaming Yang, Yue Xiao, Jie Yang, Xin Wang, Ran Zhang, Xujun Zhang, Xinyu Shen, Nan Zhou

**Affiliations:** 1 Key Laboratory of Environmental Medicine and Engineering of Ministry of Education, Department of Epidemiology and Health Statistics, School of Public Health, Southeast University, Nanjing 210009, China; 220234013@seu.edu.cn (T.X.); xjzhang@seu.edu.cn (X.Z.); 18852887887@163.com (X.S.); 2 Yixing Center for Disease Control and Prevention, Yixing 214200, China; yxyyming@163.com (Y.Y.); xy.ybb@163.com (Y.X.); 3 Jiangsu Provincial Center for Disease Control and Prevention, Nanjing 210009, China; july-summer@jscdc.cn (J.Y.); wx1995@connect.hku.hk (X.W.); 4 School of Public Health, Nanjing Medical University, Nanjing 211166, China; 5 Department of Mental Health, School of Public Health, Nanjing Medical University, Nanjing 211166, China; ran_1994@yeah.net

**Keywords:** comorbid anxiety and depression, internet addiction, adolescent, latent profile analysis, network analysis

## Abstract

**Highlights:**

**What are the main findings?**

- Our findings revealed the association between Internet addiction and comorbid anxiety and depression.
- Comorbidity networks of anxiety and depression differed across Internet addiction profiles (*“regular”* profile, *“risk”* profile, and *“addiction”* profile), indicating profile-specific symptom architectures.

**What are the implications of the main findings?**

- Screening and prevention efforts should prioritize children and adolescents in the *“risk”* and *“addiction”* profiles.
- Targeting key symptoms such as “Downhearted and blue”, “Close to panic”, “Lack of motivation”, and “Meaninglessness of life” may help reduce the risk of comorbid anxiety and depression among children and adolescents with excessive Internet use.

**Abstract:**

**Objectives**: This study aims to examine Internet addiction profiles, their associations with comorbid anxiety and depression, and characterize network architectures of anxiety and depression across profiles. **Methods**: From November 2022 to November 2023, we conducted a short-term cohort study including 2503 students. Latent profile analysis (LPA) and multinomial logistic regression analysis were employed to investigate the association between Internet addiction and comorbidity of anxiety and depression, and network analysis was used to characterize anxiety–depression network structure within each profile. **Results**: LPA identified three profiles of Internet addiction, which were labeled: *“regular”* (66.60%) profile, *“risk”* profile (23.09%), and *“addiction”* profile (10.31%). The incidence of comorbid anxiety and depression was 10.67%. Both the *“risk”* (adjusted OR = 1.76, 95% CI: 1.27–2.44) and *“addiction”* (adjusted OR = 2.12, 95% CI: 1.39–3.24) profiles were significantly associated with increased comorbidity risk. The “dass13” (“Downhearted and blue”) emerged as a core symptom, and “dass15” (“Close to panic”) was identified as a key bridge symptom across three network models. The edge weight for the dass05–dass21 (Lack of motivation–Meaninglessness of life) was higher in the *“risk”* profile than in the *“addiction”* profile. **Conclusions**: Children and adolescents in the *“risk”* and *“addiction”* profiles were significantly more likely to experience comorbid anxiety and depression. “dass13” (“Downhearted and blue”) and “dass15” (“Close to panic”) can be used as the key target during intervention. Targeted interventions can be implemented for children and adolescents in the *“risk”* and *“addiction”* profiles.

## 1. Introduction

Adolescence represents a critical period for psychological development, and research has shown that the emergence of psychiatric disorders in adulthood is associated with the emotional disorders observed during adolescence [1]. Anxiety and depression are the most common psychological problems among children and adolescents [2]. Studies indicate that approximately 20.5% of children and adolescents worldwide experience anxiety [3], and one-fifth of children and adolescents exhibit depression [4]. In China, surveys reveal that 26.9% of children and adolescents have anxiety and 26.17% exhibit depression [5,6]. Anxiety and depression in children and adolescents can lead to loneliness, substance abuse, psychosocial functioning, and even suicide [7,8,9].

Internet addiction is commonly defined as a loss of control over internet use and may impair psychological development and be associated with functional impairments in children and adolescents [2,10]. Numerous studies have explored the associations between Internet addiction and anxiety, as well as between Internet addiction and depression [11,12,13]. However, variable-centered approaches to evaluate Internet addiction in children and adolescents do not consider individual factors or crucial information within individuals. Recent studies have employed latent profile analysis (LPA) to identify distinct Internet addiction profiles and examine their associations with anxiety and depression. For example, a prior study used LPA to classify Internet addiction into three profiles (non-Internet addiction, low-Internet addiction, and high-Internet addiction) and found that higher levels of anxiety and depression were concentrated among children and adolescents in the high addiction profile [14]. Longitudinal research among university students has further shown that higher levels of problematic social media use and gaming prospectively predict increased anxiety and depressive symptoms [15]. Nevertheless, these studies have typically examined each profile’s impact on anxiety or depression in isolation, despite the frequent co-occurrence of anxiety and depression. Moreover, one study has found that among children and adolescents with anxiety disorders, the comorbidity rate of depression ranged from 10% to 15%, and among those with depressive disorders, the comorbid anxiety rate ranged from 15% to 75% [16]. Compared with individuals experiencing only anxiety or only depression, children and adolescents with comorbid anxiety and depression face the highest risks of adverse outcomes, such as cardiovascular disease, obesity, and substance abuse [17]. Therefore, the associations between distinct Internet addiction profiles and the comorbidity of anxiety and depression in children and adolescents should be investigated.

Traditional psychopathology frameworks assume that mental symptoms reflect an underlying disease [18]. Previous studies have primarily conceptualized anxiety and depression as latent disorders and conducted analyses on the total scores of specific scales [19]. However, this psychometric approach may obscure important differences and relationships among symptoms [20]. Network theory offers an alternative perspective. Within network analysis frameworks, interactions between symptoms are modeled as directed or undirected networks. Nodes correspond to specific symptoms and are connected via edges (e.g., positive or negative associations) [21]. In recent years, an increasing number of scholars have applied network analysis to study the anxiety and depression network in various populations, such as children and adolescents exposed to trauma and adolescent survivors of sexual abuse [22,23]. However, little attention has been paid to the structure of the anxiety and depression network among children and adolescents with distinct Internet addiction profiles, which should be examined to identify the key symptoms within each network.

In summary, this study aims to (a) identify distinct Internet addiction profiles using LPA, (b) examine associations between these profiles and anxiety–depression comorbidity, and (c) characterize network architectures across Internet addiction profiles by network analysis.

## 2. Materials and Methods

### 2.1. Design and Participants

This short-term cohort study was conducted between November 2022 and November 2023. A single follow-up assessment was administered 12 months after baseline, and enrolled students from Jiangsu Province, China. Participants were recruited from four schools, and 2595 students were invited to participate. Students were excluded if they: (1) scored >7 on the anxiety subscale or >9 on the depression subscale of the 21-item Depression Anxiety Stress Scale (DASS-21); (2) had a history of psychiatric disorders; (3) declined to participate. The outcome was defined as new-onset comorbidity, referring to the simultaneous new emergence of both anxiety and depression in individuals who scored below the cutoff threshold for both anxiety and depression scales at baseline. The baseline questionnaire collected data on demographic characteristics, peer relationship problems, food addiction, and depression and anxiety status. Ultimately, 2503 children and adolescents were included in the study, and they provided valid questionnaires at both baseline and follow-up assessments.

### 2.2. Measurements

#### 2.2.1. Internet Addiction

At baseline, the Revised Chen Internet Addiction Scale (CIAS-R) [24] was used to assess Internet addiction in children and adolescents. The CIAS-R comprises 26 self-report items designed to assess five dimensions: compulsive use, withdrawal, tolerance, interpersonal health problems and time management problems on a four-point Likert scale ranging from “1 = does not describe my experience” to “4 = fully describes my experience”. The total CIAS-R scores range from 26 to 104. CIAS-R scores ≥ 57 were classified as Internet addiction [25]. The scale has demonstrated excellent internal consistency in this study with a Cronbach’s alpha of 0.90.

#### 2.2.2. Depression and Anxiety

At both baseline and follow-up, anxiety and depression of participants were measured using the DASS-21 [26]. Each subscale contains seven items. Items are rated on a four-point Likert scale from 0 (“did not apply to me at all”) to 3 (“applied to me very much or most of the time”). Scores of >9 on the depression subscale indicate depression, scores of >7 on the anxiety subscale denote anxiety, and scores exceeding both thresholds indicate comorbid anxiety and depression. The scale has been widely used to measure anxiety and depression in Chinese population [27,28]. The subscales showed excellent internal consistency in this study, with Cronbach’s alphas of 0.90 for anxiety and 0.85 for depression.

#### 2.2.3. Other Measurement

At baseline, sociodemographic variables were collected via a self-designed questionnaire, including gender, educational stage, only child status, boarding status, student cadre experience, and family type. Maternal education level was categorized as low (primary school or below), middle (junior or senior high school), or high (college or above). Simultaneously, peer relationship problems were assessed with the Strengths and Difficulties Questionnaire (SDQ) [29,30]. Each of the five items was rated as 0 (not true), 1 (somewhat true), or 2 (certainly true), yielding a total score ranging from 0 to 10. Scores were categorized as normal (0–3), borderline (4–5), or abnormal (6–10). Food addiction was measured using modified Yale Food Addiction Scale 2.0 (mYFAS 2.0) [31]. The scale comprised 11 items assessed on an 8-point scale ranging from 0 (never) to 7 (daily). Each item was scored as 0 (threshold not met) or 1 (threshold met), yielding a symptom count score ranging from 0 to 11. Participants were classified as absent (symptom count <2) or present (symptom count ≥ 2). Family conflict was evaluated using the Family Environment Scale (FES) [32]. The subscale comprised 9 items, each rated as 0 (yes) or 1 (no), yielding a total score ranging from 0 to 9. Scores were categorized as low conflict (0–1), moderate conflict (2–5), or high conflict (6–9).

#### 2.2.4. Latent Profile Analysis

LPA was performed using Mplus (version 8.3) to identify Internet addiction profiles among children and adolescents. The five dimensions of CIAS-R were used as profile indicators. Seven statistics were used to determine the optimal number of latent profiles: Akaike information criterion (AIC), Bayesian information criterion (BIC), sample-size-adjusted BIC (aBIC), Lo–Mendell–Rubin likelihood ratio test (LMRT), bootstrap likelihood ratio test (BLRT), entropy, and size of the smallest profile. Lower values of AIC, BIC, and aBIC indicate better model fit. Significant LMRT and BLRT results (*p* < 0.05) suggest that a model with k profiles fits the data better than one with k-1 profiles [33]. Higher entropy values reflect more accurate classification. The smallest profile should comprise at least 5% of the sample [34].

#### 2.2.5. Descriptive Analysis and Logistic Analysis

Baseline characteristics were described using median (Interquartile Range, IQR) for age and categorical variables were presented as percentages according to Internet addiction profiles. Statistical comparisons of comorbidity incidence between profiles were performed using chi-square (χ^2^) tests or Fisher’s exact tests.

Multivariate logistic regression models were used to examine the association between Internet addiction profiles and comorbid depression and anxiety. The outcomes were constructed as new-onset comorbid anxiety and depression at follow-up, with participants classified as either having developed comorbidity (comorbidity present) or not (comorbidity absent). The exposure variable was Internet addiction profile at baseline (regular, risk, and addiction), entered as a categorical variable with the regular profile as the reference group. Covariates were selected based on the I-PACE framework [35] and prior empirical evidence. Standard sociodemographic variables—gender [36,37,38], educational stage [39,40,41], only child status [42,43,44], family structure [45,46], student cadre experience [47,48], boarding status [49,50], maternal education level [51,52], baseline anxiety and depression levels—were included as baseline controls given their established associations with adolescent Internet use patterns and internalizing outcomes in prior research. Beyond sociodemographic factors, the following covariates were included based on specific theoretical and empirical rationale: peer relationship problems were adjusted for as a psychosocial confounder, as poor peer relationships have been independently linked to both problematic Internet use and adolescent anxiety and depression [53,54]. Food addiction was included as a behavioral confounder, given evidence that it shares common impulsivity and emotion dysregulation pathways with Internet addiction and is associated with internalizing symptoms [55,56]. Family conflict was included as a family-context confounder, as family relational stress has been associated with both adolescent Internet addiction and anxiety–depression outcomes [57,58]. Baseline anxiety and depression scores were entered as continuous variables. Data were analyzed using R (version 4.3.3). Results were presented using odds ratio (OR) and 95% confidence intervals (CI). In all analyses, *p*-values (two-sided) less than 0.05 were considered statistically significant.

#### 2.2.6. Network Analysis

Anxiety and depression networks across Internet addiction profiles were estimated using graphical lasso regularization (gamma = 0.5) via the R “bootnet” package (version 1.6.5). The graphical lasso with EBIC model selection was chosen as it produces sparse, interpretable networks by shrinking small spurious edges to zero, which is appropriate for psychological symptom data. A gamma value of 0.5 was selected as it represents a moderate sparsity penalty that balances parsimony with sensitivity and has been recommended as the default setting in psychological network analysis [59]. The “qgraph” package (version 1.6.5) was used to visualize each network, employing an identical layout across profiles to facilitate direct visual comparison. Due to the ordinal and non-normal nature of the data, Spearman’s rank correlation was used to compute the association matrix [60]. Although polychoric correlations are sometimes preferred for ordinal data, Spearman correlations have been shown to perform comparably in network estimation and are more computationally stable in smaller subgroup samples. Expected influence (EI) and bridge expected influence (BEI) were calculated to identify central and bridge symptoms within each network [61]. EI was selected in preference to node strength or betweenness centrality as it accounts for the sign of edge weights and has been recommended as a more valid centrality index when networks contain both positive and negative edges [62]. Edge weight accuracy was assessed via non-parametric bootstrapping (1000 iterations) using the R package “bootnet” (v1.6.5), with accuracy evaluated by examining the width of 95% bootstrap confidence intervals around each edge weight estimate. The stability of centrality indices was evaluated using the case-dropping subset bootstrap with 1000 iterations; a CS-coefficient ≥ 0.25 was considered acceptable and ≥0.50 was considered good [63].

Finally, bootstrap difference test was performed to evaluate differences in edge weights and centrality indices between nodes [37]. The R package “Network Comparison Test” 2.0.1 was used to evaluate differences in global strength and network structure, with Bonferroni correction applied for multiple comparisons [64].

## 3. Results

### 3.1. Sample Characteristics and Internet Addiction Profile

A total of 2503 participants were included in this study. Table 1 reports the characteristics of participants. The median age of participants at baseline was 13.90 (IQR: 12.30–16.20) years old. Of 2503 participants, nearly half of the students (48.86%, 1223/2503) were female. The majority of participants had siblings (66.44%, 1663/2503), came from an intact family (81.58%, 2042/2503), were non-boarding (85.86%, 2149/2503), had no food addiction symptoms (96.76%, 2422/2503), reported normal peer relationships (76.03%, 1903/2503), and had mothers with middle education level (41.91%, 1049/2503).

The model fit statistics of LPA are summarized in Table S1. The three-profile solution had relatively lower AIC, BIC, and aBIC, and the BLRT was <0.01; moreover, the three-profile model yielded the highest entropy (Table S1). The additional profile in the four-profile solution appeared to be a minor quantitative split of an existing class rather than a qualitatively distinct pattern, offering limited theoretical added value. Guided by parsimony and theoretical interpretability, the three-profile model was selected.

**Figure 1:**
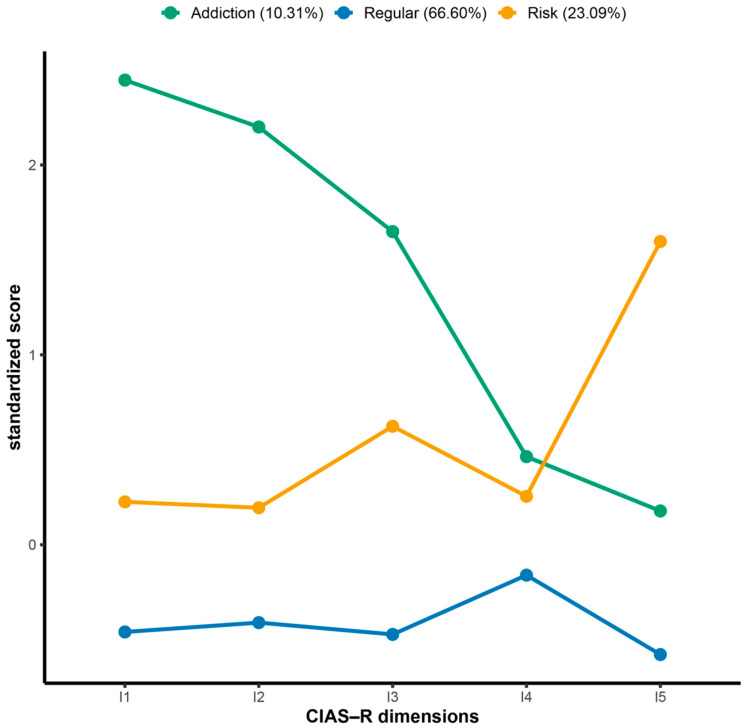
Each dimension standardized score of CIAS-R for three-profile solution. I1: Compulsive use; I2: Withdrawal; I3: Tolerance; I4: Interpersonal health problems; I5: Time management problems.

Regarding profile characteristics, Profile 1 scored significantly lower than Profiles 2 and 3 across all dimensions as well as on the mean total CIAS-R score (M = 39.64), and was labeled as the *“regular”* profile (66.6 0%, *n* = 1667). Profile 2 was characterized by a relatively elevated time-management problem dimension compared with Profiles 1 and 3; however, it scored significantly lower than Profile 3 on the mean total CIAS-R score (M = 51.13). This profile was labeled as the *“risk”* profile (23.09%, *n* = 578). Profile 3 scored higher than the other two profiles on the first four dimensions and also exhibited the highest mean total CIAS-R score (M = 65.57), and was therefore labeled as the *“addiction”* profile (10.31%, *n* = 258) (Figure 1).

The distribution of educational stage (*p* < 0.001), type of family (*p* < 0.05), student cadre experience (*p* < 0.05), boarding status (*p* < 0.001), food addiction (*p* < 0.001), Family structure (*p* < 0.001), maternal education level (*p* < 0.001), and peer relationship (*p* < 0.001) differed significantly across Internet addiction profiles. Children and adolescents at higher educational stages, those with boarding-school attendance, food-addiction symptoms, no student cadre experience, non-intact families, higher levels of family conflict, lower maternal education level or poorer peer relationships were more likely to be classified into the *“risk”* profile or *“addiction”* profile (Table 1).

### 3.2. Association Between Internet Addiction and Comorbidity of Depression and Anxiety

The incidence of comorbid depression and anxiety was 10.67% (267/2503), while 11.43% (286/2503) of the sample suffered from single morbidity (5.91% anxiety only, 148/2503; 5.51% depression only, 138/2503). Within the *“regular”* profile, the incidence of comorbid anxiety and depression was 7.02% (117/1667); in the *“risk”* profile, it was 16.61% (96/578); and in the *“addiction”* profile, it was 20.93% (54/258). After adjusting for covariates in the model, children and adolescents from the *“risk”* profile and *“addiction”* profile remained more likely to have comorbid anxiety and depression compared to those from the *“regular”* profile (adjusted OR = 1.76, 95% CI [1.27, 2.44] for *“risk”* profile; adjusted OR = 2.12, 95% CI [1.39, 3.24] for *“addiction”* profile) (Table 2).

### 3.3. Network Analysis Results

In the anxiety and depression network of the *“regular”* profile, the centrality stability coefficient (CS-C) values were 0.517 for EI and 0.595 for BEI. In the *“risk”* profile network, CS-C values of the EI and BEI were 0.361 and 0.595, respectively (Figures S1 and S2). In the *“addiction”* profile network, the CS-C values were 0.593 for both EI and BEI. Confidence intervals for edge weights were small to moderate across all three networks (Figure S3).

**Figure 2:**
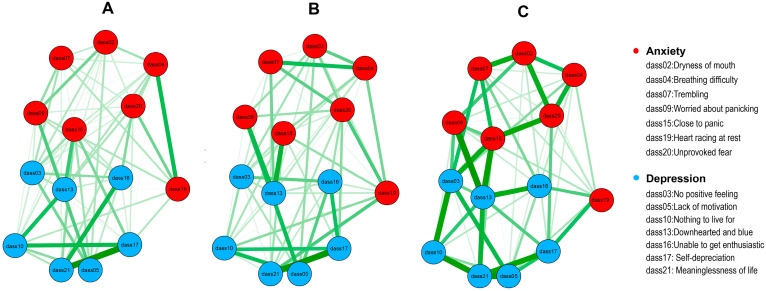
Network models of anxiety and depression, green edges represent positive correlations, red edges represent negative correlations. (**A**) Network model of the *“regular”* profile; (**B**) Network model of the *“risk”* profile; (**C**) Network model of the *“addiction”* profile.

**Figure 3:**
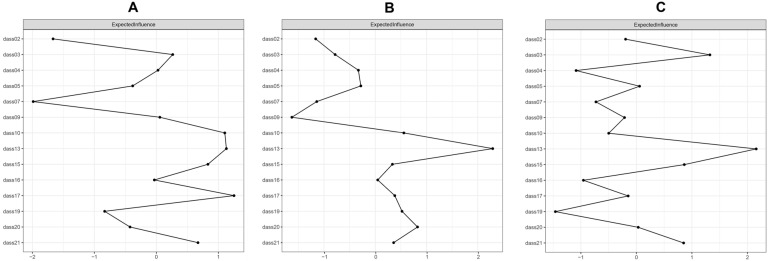
Expected influence of network nodes. (**A**) Expected influence of the *“regular”* profile; (**B**) Expected influence of the *“risk”* profile; (**C**) Expected influence of the *“addiction”* profile.

**Figure 4:**
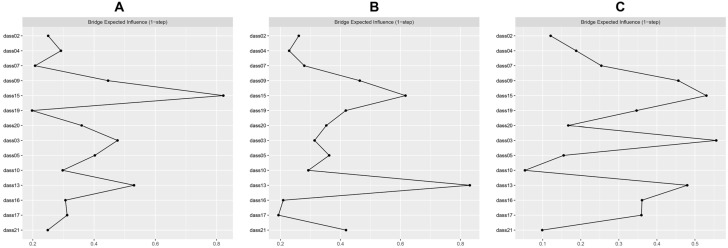
Bridge expected influence of network nodes. (**A**) Bridge expected influence of the *“regular”* profile; (**B**) Bridge expected influence of the *“risk”* profile; (**C**) Bridge expected influence of the *“addiction”* profile.

Network models of anxiety and depression among the *“regular”* profile, *“risk”* profile, and *“addiction”* profile are shown in Figure 2. The network of the *“regular”* profile comprised 14 nodes and 72 non-zero edges of 91 possible edges (79.12%). The network of the *“risk”* profile comprised 14 nodes and 69 non-zero edges of 91 possible edges (75.82%). The network of the *“addiction”* profile comprised 14 nodes and 69 non-zero edges of 91 possible edges (75.82%). Tables S2–S4 illustrate edge weights for these three groups. Most edge weight differences were not statistically significant (Figure S4). EI values are presented in Figure 3. In the *“regular”* profile, dass17 (“Self-depreciation”) ranked highest, followed by dass13 (“Downhearted and blue”) and dass10 (“Nothing to live for”). In the *“risk”* profile, dass13 (“Downhearted and blue”) ranked highest, followed by dass20 (“Unprovoked fear”) and dass10 (“Nothing to live for”). In the *“addiction”* profile, dass13 (“Downhearted and blue”) ranked highest, followed by dass03 (“No positive feeling”) and dass15 (“Close to panic”). These symptoms consistently demonstrated higher EI values than most others (Figure S5). BEI values across the three profiles are shown in Figure 4. Dass13 (“Downhearted and blue”) and dass15 (“Close to panic”) emerged as prominent bridge symptoms across all three profiles. In the *“regular”* profile, dass03 (“No positive feeling”) was also a key bridge symptom. In the *“risk”* profile, dass09 (“Worried about panicking”) was additionally identified as a prominent bridge symptom. In the *“addiction”* profile, dass03 (“No positive feeling”) was also identified as a prominent bridge symptom. These symptoms exhibited significantly higher bridge centrality compared to most others (Figure S6).

The *“risk”* profile—*“addiction”* profile comparison indicated a structural difference (M = 0.31, *p* < 0.05), with the largest edge discrepancy for dass05–dass21 (Lack of motivation-Meaninglessness of life), stronger in the *“risk”* profile. Other pairwise comparisons showed no significant differences in global strength or network structure (Figures S7 and S8).

## 4. Discussion

The three Internet addiction profiles identified in this study are consistent with previous findings [14,65]. However, our findings differ from those of Wang et al. [12], who reported a fourth “low addiction” profile; our study did not identify this group, possibly because Wang’s study included children under 10 years old, whereas our sample did not.

Our results showed that the *“risk”* and *“addiction”* profiles were associated with a significantly higher likelihood of comorbid anxiety and depression than the *“regular”* profile. Consistent with our regression findings, some studies suggest that higher levels of Internet addiction or problematic social media use confer increased risk for depression and anxiety in adolescence [15,66]. Several theoretical models help explain why problematic Internet use may be associated with the subsequent emergence of anxiety and depression. Within the I-PACE framework, children and adolescents under stress or low mood may increasingly rely on online activities for short-term mood regulation and escape. In turn, this reliance on online mood regulation may be associated with greater emotion dysregulation and stress sensitivity, thereby increasing the likelihood of subsequent anxiety and depressive symptoms [35,67]. Excessive Internet use reduces time available for exercise, and physically inactive individuals are more likely to experience depression and anxiety than those who exercise regularly [68]. Additionally, low activity is linked to poor sleep quality, which may contribute to generalized anxiety disorder (GAD) and, subsequently, major depressive disorder [69,70]. Thus, reduced engagement in offline rewarding activities (e.g., exercise, hobbies, in-person friendships) and cumulative sleep disruption may represent plausible pathways linking problematic Internet use to the co-occurrence and progressive worsening of anxiety and depression symptoms.

“Downhearted and blue” (dass13) showed consistently high expected influence across all three profiles. This is in line with previous studies [71,72] and suggests that low mood is a key node in children and adolescents’ network of anxiety and depression symptoms. In addition, “Close to panic” (dass15) ranked among the top three bridge symptoms across profiles, consistent with prior network findings that panic-related symptoms are among the most important bridges between anxiety and depression [73]. This indicates that panic-related arousal may efficiently link anxiety and depressive symptom clusters. The bridge role of dass15 (“Close to panic”) suggests that panic-related arousal may serve as an early warning signal linking anxiety to depression, and its routine assessment among adolescents with problematic Internet use may support timely identification of comorbidity risk. Given the consistently high expected influence of dass13 (“Downhearted and blue”) across all three profiles, embedding brief low-mood monitoring into school health checks, particularly for students identified in the “risk” or “addiction” profiles, could facilitate earlier detection of at-risk individuals before full comorbid episodes emerge [74,75].

We further observed a shift in centrality across profiles. In the *“regular”* profile, “Self-depreciation” (dass17) had the highest centrality, whereas in the *“addiction”* profile, “No positive feeling” (dass03) rose to the second-highest position. This pattern suggests that children and adolescents with Internet addiction may be more likely to experience anhedonia, consistent with the fact that addictive behaviors are associated with altered reward functioning [76]. Supporting this interpretation, previous research reported more severe anhedonia symptoms among individuals with problematic Internet use [77].

The dass05–dass21 connection was significantly stronger in the *“risk”* profile. One plausible explanation is that time-management problems related to excessive Internet use may be associated with accumulated real-life difficulties, which in turn may reinforce the belief that effort is meaningless, strengthening this cognitive–motivational link [78]. However, this implication should be interpreted cautiously. Because many edge differences were not statistically significant, the identified profile-specific edges (e.g., the dass05–dass21 link in the “risk” profile) should be viewed as localized variations rather than as indicating a complete structural reorganization of the network. This edge may nonetheless serve as an early intervention target, particularly for adolescents in the “risk” profile. Meaning-centered or values-based approaches, such as Acceptance and Commitment Therapy (ACT) adapted for adolescents, may be worth exploring in this context, given their potential to address both motivational deficits and sense of meaninglessness. School counselors working with students showing early signs of problematic Internet use and time-management difficulties could consider incorporating brief values clarification and committed action exercises into existing support frameworks, such as routine counseling sessions or peer support programs, to help adolescents reconnect with personally meaningful goals [79,80].

Finally, several limitations should be acknowledged. First, participants were recruited from four schools in Jiangsu Province, which restricts the geographic and sociocultural representativeness of the findings. Second, students with baseline DASS-21 anxiety scores > 7 or depression scores > 9 were excluded to enable new-onset case identification. While this design decision ensures that the observed outcome reflects new-onset comorbidity rather than the continuation or progression of pre-existing symptoms, it means that the analytic sample represents a selected, below-threshold subgroup. The observed comorbidity incidence should not be interpreted as representative of the true incidence among all adolescents, and the associations reported here may not generalize to individuals with pre-existing symptoms. or to populations from other geographic and sociocultural contexts. Third, the study comprised only two assessment time points, and findings should not be interpreted as evidence of a full longitudinal characterization of these relationships. Future studies with longer follow-up periods and more advanced modeling approaches are warranted.

## 5. Conclusions

In conclusion, this study identified three distinct Internet addiction profiles among Chinese children and adolescents through LPA: the *“regular”* profile, the *“risk”* profile, and the *“addiction”* profile. Children and adolescents in the *“risk”* and *“addiction”* profiles were significantly more likely to experience comorbid anxiety and depression than those in the *“regular”* profile, with the *“addiction”* profile exhibiting the strongest association. Network analyses revealed that in the three profiles, “Close to panic” and “No positive feeling” were key bridge symptoms. The Lack of motivation–Meaninglessness of life link was a key edge in the *“risk”* profile. These findings underscore the importance of early identification and intervention for children and adolescents at risk of Internet addiction and highlight specific symptoms that may serve as therapeutic targets for reducing the risk of comorbid anxiety and depression in this population.

## Figures and Tables

**Table 1:** Baseline characteristics of 2503 participants by Internet addiction status.

Factors	Total	*“Regular” Profile*	*“Risk” Profile*	*“Addiction” Profile*	Overall Comparison
	*n* = 2503	*n* = 1667	*n* = 578	*n* = 258	
**Gender**					*p* > 0.05
Male	1280 (51.14%)	843 (50.57%)	298 (51.56%)	139 (53.88%)	
Female	1223 (48.86%)	824 (49.43%)	280 (48.44%)	119 (46.12%)	
**Educational stage**					*p* < 0.001
Primary school	912 (36.44%)	740 (44.39%)	112 (19.38%)	60 (23.26%)	
Middle school	954 (38.11%)	675 (40.49%)	213 (36.85%)	66 (25.58%)	
High school	637 (25.45%)	252 (15.12%)	253 (43.77%)	132 (51.16%)	
**Only child**					*p* > 0.05
Had siblings	1663 (66.44%)	1121 (67.25%)	371 (64.19%)	171 (66.28%)	
Only child	840 (33.56%)	546 (32.75%)	207 (35.81%)	87 (33.72%)	
**Family structure**					*p* < 0.05
Intact families	2042 (81.58%)	1379 (82.72%)	470 (81.31%)	193 (74.81%)	
Non-intact families	461 (18.42%)	288 (17.28%)	108 (18.69%)	65 (25.19%)	
**Student cadre**					*p* < 0.05
No	1247 (49.82%)	803 (48.17%)	314 (54.33%)	130 (50.39%)	
Yes	1256 (50.18%)	864 (51.83%)	264 (45.67%)	128 (49.61%)	
**Boarding status**					*p* < 0.001
No	2149 (85.86%)	1485 (89.08%)	464 (80.28%)	200 (77.52%)	
Yes	354 (14.14%)	182 (10.92%)	114 (19.72%)	58 (22.48%)	
**Food addiction**					*p* < 0.001
No	2422 (96.76%)	1649 (98.92%)	554 (95.85%)	219 (84.88%)	
Yes	81 (3.24%)	18 (1.08%)	24 (4.15%)	39 (15.12%)	
**Family conflict**					*p* < 0.001
Low	1469 (58.69%)	1057 (63.41%)	316 (54.67%)	96 (37.21%)	
Moderately	966 (38.59%)	582 (34.91%)	242 (41.87%)	142 (55.04%)	
High	68 (2.72%)	28 (1.68%)	20 (3.46%)	20 (7.75%)	
**Peer relationship**					*p* < 0.001
Normal	1903 (76.03%)	1295 (77.68%)	466 (80.62%)	142 (55.04%)	
Fringe	487 (19.46%)	319 (19.14%)	90 (15.57%)	78 (30.23%)	
Abnormal	113 (4.51%)	53 (3.18%)	22 (3.81%)	38 (14.73%)	
**Maternal education level**					
low	637 (25.45%)	371 (22.26%)	171 (29.58%)	95 (36.82%)	*p* < 0.001
middle	1049 (41.91%)	716 (42.95%)	229 (39.62%)	104 (40.31%)	
high	817 (32.64%)	580 (34.79%)	178 (30.80%)	59 (22.87%)	

**Table 2:** Results of logistic regression analysis.

Exposure	Unadjusted Odds Ratio (95% CI)	*p* Value	Adjusted Odds Ratio (95% CI)	*p* Value
*“regular”* profile	Reference	-	Reference	-
*“risk”* profile	2.80 (2.09–3.74)	<0.001	1.76 (1.27–2.44)	<0.05
*“addiction”* profile	3.99 (2.78–5.73)	<0.001	2.12 (1.39–3.24)	<0.05

## Data Availability

The data presented in this study are available on request from the corresponding author due to privacy and ethical restrictions involving children and adolescents.

## Supplementary Materials

The following supporting information can be downloaded at: https://www.mdpi.com/article/10.3390/healthcare14070862/s1, Figure S1: Stability of expected influence by case dropping sub-set bootstrap of network among *“regular”* profile, *“risk”* profile and *“addiction”* profile; Figure S2: Stability of bridge expected influence by case dropping sub-set bootstrap of *“regular”*, *“risk”* profile and *“addiction”* profile; Figure S3: Bootstrapped confidence intervals of edge weights; Table S1: Latent Profile Model Fit Indices; Table S2: Estimated edge weights of the *“regular”* profile; Table S3: Estimated edge weights of the *“risk”* profile; Table S4: Estimated edge weights of the *“addiction”* profile; Figure S4: Estimation of edge weight difference by bootstrapped difference test; Figure S5: Estimation of node expected influence difference by bootstrapped difference test; Figure S6: Estimation of node bridge expected influence difference by bootstrapped difference test; Figure S7: Comparison of network global strength; Figure S8: Comparison of network structure.

## Abbreviations

The following abbreviations are used in this manuscript:

CIAS-R	Revised Chen Internet Addiction Scale
M	Mean
IQR	Interquartile Range
CS-C	centrality stability coefficient
EI	Expected influence
BEI	bridge expected influence
