# Supplementary material for: Association Between Internet Addiction and Comorbid Anxiety and Depression in Chinese Children and Adolescents: A Latent Profile Analysis and Network Analysis
Source: Healthcare (Basel). 2026 Mar 27;14(7):862. doi: 10.3390/healthcare14070862 (PMC13074099; doi:10.3390/healthcare14070862)
Supplement: Supplementary file 1 [file healthcare-14-00862-s001.zip › healthcare-4182072-supplementary.pdf]

Supplementary material

Table S1. Latent Profile Model Fit Indices

Figure S1. Stability of Expected Influence by case dropping sub-set bootstrap of network among *"regular"* profile, *"risk"* profile and *"addiction"* profile

Figure S2. Stability of bridge Expected Influence by case dropping sub-set bootstrap of *"regular"*, *"risk"* profile and *"addiction"* profile

Figure S3. Bootstrapped confidence intervals of edge weights

Table S2. Estimated edge weights of the *"regular"* profile

Table S3. Estimated edge weights of the *"risk"* profile

Table S4. Estimated edge weights of the *"addiction"* profile

Figure S4. Estimation of edge weight difference by bootstrapped difference test

Figure S5. Estimation of node Expected Influence difference by bootstrapped difference test

Figure S6. Estimation of node bridge Expected Influence difference by bootstrapped difference test

Figure S7. Comparison of network global strength

Figure S8. Comparison of network structure

Table S1. Latent Profile Model Fit Indices

Model	AIC	BIC	aBIC	Entropy	LMRT(P)	BLRT(P)
Model 1	35531.03	35589.28	35557.51	—	—	—
Model 2	30639.18	30732.39	30681.55	0.97	<0.01	<0.01
**Model 3[a]**	**27317.24**	**27445.40**	**27375.50**	**0.98**	**<0.01**	**<0.01**
Model 4	26166.23	26329.33	26240.37	0.96	<0.01	<0.01

AIC = Akaike Information Criteria; BIC = Bayesian Information Criteria; aBIC = adjusted Bayesian Information Criterion; LMRT = LoMendell-Rubin adjusted likelihood ratio test; BLRT = Bootstrap likelihood ratio test.

[a] The best fit solution is indicated in bold.

Figure S1. Stability of Expected Influence by case dropping sub-set bootstrap of network among *"regular"* profile, *"risk"* profile and *"addiction"* profile.

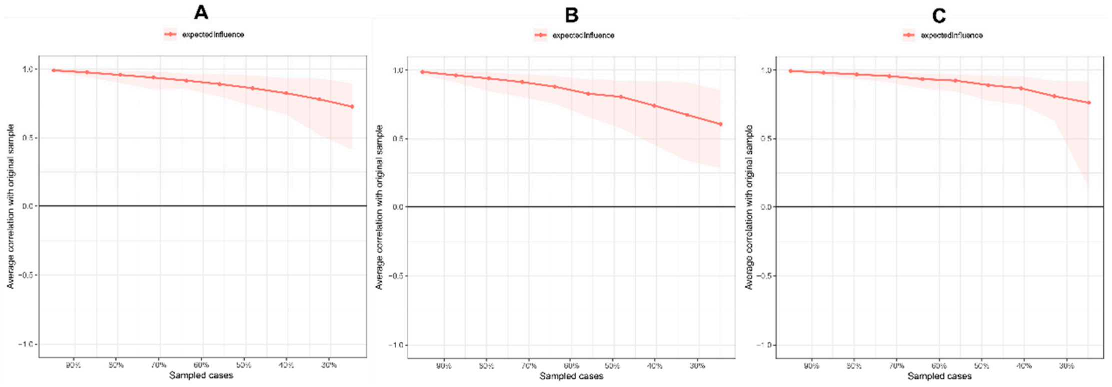

Note: A percentage of the original sample cases is indicated on the x-axis at each step. The y-axis represents a correlation between the centrality indices of the original network and those of the re-estimated networks after dropping increasing percentages of cases.

A: Stability of Expected Influence of *"regular"* profile; B: Stability of Expected Influence of *"risk"* profile; C: Stability of Expected Influence of *"addiction"* profile.

Figure S2. Stability of bridge Expected Influence by case dropping sub-set bootstrap of *"regular"*, *"risk"* profile and *"addiction"* profile.

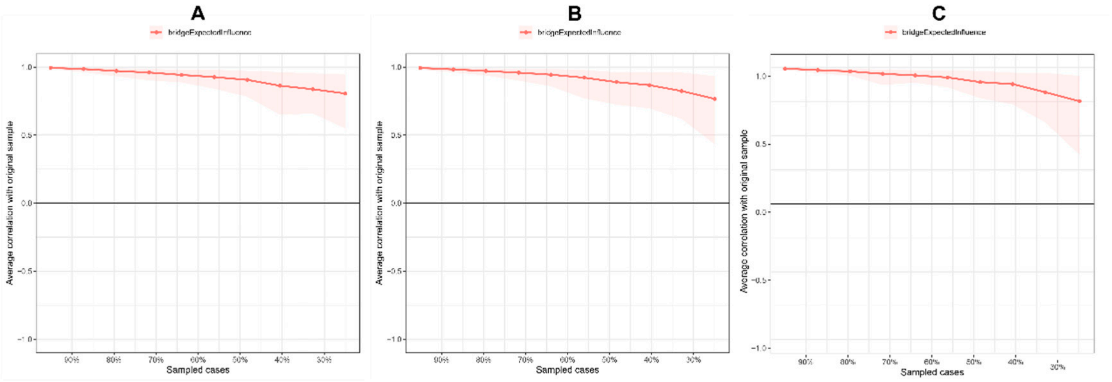

Note: A percentage of the original sample cases is indicated on the x-axis at each step. The y-axis represents a correlation between the centrality indices of the original network and those of the re-estimated networks after dropping increasing percentages of cases. Correlations between EI and bridge EI are shown on the line, and 95 % CIs are indicated on the area.

A: Stability of bridge Expected Influence of *"regular"* profile; B: Stability of bridge Expected Influence of pre-augmentation *"risk"* profile; C: Stability of bridge Expected Influence of post-augmentation *"addiction"* profile.

Figure S3. Bootstrapped confidence intervals of edge weights

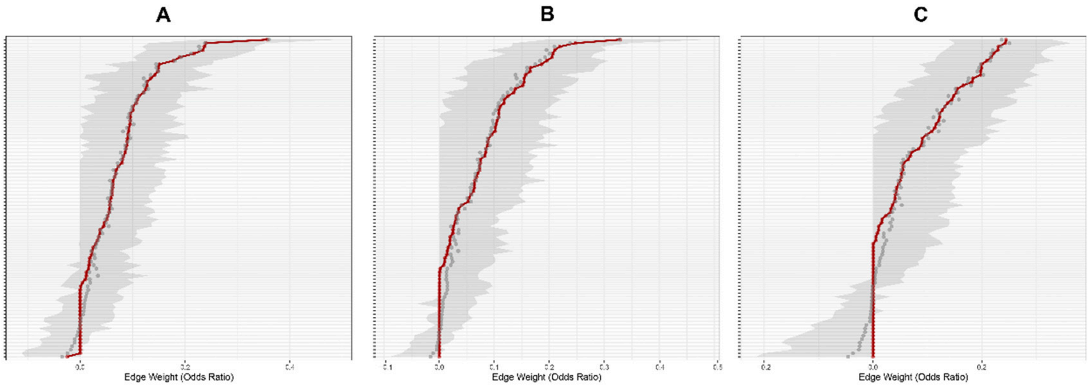

Note: The red dots indicate the value of each edge weight, arranged from highest value to lowest value. Using the non-parametric bootstrap procedure, the grey area represents the 95% Confidence Intervals of edge weights.

A: confidence intervals of edge weights of *"regular"*profile; B: confidence intervals of edge weights of *"risk"*profile; C: confidence intervals of edge weights of *"addiction"*profile.

Table S2. Estimated edge weights of the *"regular"* profile

	dass02	dass04	dass07	dass09	dass15	dass19	dass20	dass03	dass05	dass10	dass13	dass16	dass17	dass21
dass02	0.00	0.11	0.07	0.15	0.02	0.01	0.06	0.09	0.10	0.04	0.05	0.00	0.00	-0.02
dass04	0.11	0.00	0.06	0.02	0.07	0.23	0.10	0.06	0.02	0.01	0.09	0.00	0.02	0.09
dass07	0.07	0.06	0.00	0.12	0.03	0.09	0.06	0.10	0.00	0.06	0.00	0.00	0.00	0.05
dass09	0.15	0.02	0.12	0.00	0.00	0.09	0.06	0.06	0.18	0.04	0.08	0.09	0.00	0.00
dass15	0.02	0.07	0.03	0.00	0.00	0.00	0.03	0.11	0.11	0.07	0.19	0.08	0.14	0.13
dass19	0.01	0.23	0.09	0.09	0.00	0.00	0.15	0.00	0.00	0.09	0.02	0.00	0.09	0.00
dass20	0.06	0.10	0.06	0.06	0.03	0.15	0.00	0.06	0.00	0.00	0.10	0.13	0.06	0.01
dass03	0.09	0.06	0.10	0.06	0.11	0.00	0.06	0.00	0.15	0.04	0.10	0.13	0.03	0.00
dass05	0.10	0.02	0.00	0.18	0.11	0.00	0.00	0.15	0.00	0.13	0.06	0.08	0.00	0.02
dass10	0.04	0.01	0.06	0.04	0.07	0.09	0.00	0.04	0.13	0.00	0.22	0.00	0.24	0.09
dass13	0.05	0.09	0.00	0.08	0.19	0.02	0.10	0.10	0.06	0.22	0.00	0.07	0.04	0.00
dass16	0.00	0.00	0.00	0.09	0.08	0.00	0.13	0.13	0.08	0.00	0.07	0.00	0.05	0.24
dass17	0.00	0.02	0.00	0.00	0.14	0.09	0.06	0.03	0.00	0.24	0.04	0.05	0.00	0.36
dass21	-0.02	0.09	0.05	0.00	0.13	0.00	0.01	0.00	0.02	0.09	0.00	0.24	0.36	0.00

Note: dass02: Dryness of mouth; dass03: No positive feeling; dass04: Breathing difficulty; dass05: Lack of motivation; dass07: Trembling; dass09: Worried about panicking; dass10: Nothing to live for; dass13: Downhearted and blue; dass15: Close to panic; dass16: Unable to get enthusiastic; dass17: Self-depreciation; dass19: Heart racing at rest; dass20: Unprovoked fear; dass21: Meaninglessness of life.

Table S3. Estimated edge weights of the *"risk"* profile

	dass02	dass04	dass07	dass09	dass15	dass19	dass20	dass03	dass05	dass10	dass13	dass16	dass17	dass21
dass02	0.00	0.15	0.04	0.10	0.00	0.07	0.12	0.09	0.11	0.00	0.06	0.00	0.00	0.00
dass04	0.15	0.00	0.21	0.00	0.11	0.14	0.00	0.07	0.03	0.04	0.07	0.01	0.00	0.00
dass07	0.04	0.21	0.00	0.03	0.02	0.02	0.15	0.06	0.00	0.02	0.14	0.00	0.00	0.06
dass09	0.10	0.00	0.03	0.00	0.00	0.00	0.10	0.00	0.21	0.03	0.22	0.01	0.00	0.00
dass15	0.00	0.11	0.02	0.00	0.00	0.11	0.05	0.08	0.00	0.09	0.25	0.02	0.08	0.10
dass19	0.07	0.14	0.02	0.00	0.11	0.00	0.17	0.00	0.00	0.12	0.07	0.06	0.00	0.16
dass20	0.12	0.00	0.15	0.10	0.05	0.17	0.00	0.00	0.02	0.00	0.02	0.10	0.12	0.09
dass03	0.09	0.07	0.06	0.00	0.08	0.00	0.00	0.00	0.09	0.11	0.08	0.15	0.01	0.03
dass05	0.11	0.03	0.00	0.21	0.00	0.00	0.02	0.09	0.00	0.21	0.03	0.16	0.00	0.00
dass10	0.00	0.04	0.02	0.03	0.09	0.12	0.00	0.11	0.21	0.00	0.07	0.02	0.19	0.05
dass13	0.06	0.07	0.14	0.22	0.25	0.07	0.02	0.08	0.03	0.07	0.00	0.08	0.00	0.01
dass16	0.00	0.01	0.00	0.01	0.02	0.06	0.10	0.15	0.16	0.02	0.08	0.00	0.20	0.06
dass17	0.00	0.00	0.00	0.00	0.08	0.00	0.12	0.01	0.00	0.19	0.00	0.20	0.00	0.33
dass21	0.00	0.00	0.06	0.00	0.10	0.16	0.09	0.03	0.00	0.05	0.01	0.06	0.33	0.00

Note: dass02: Dryness of mouth; dass03: No positive feeling; dass04: Breathing difficulty; dass05: Lack of motivation; dass07: Trembling; dass09: Worried about panicking; dass10: Nothing to live for; dass13: Downhearted and blue; dass15: Close to panic; dass16: Unable to get enthusiastic; dass17: Self-depreciation; dass19: Heart racing at rest; dass20: Unprovoked fear; dass21: Meaninglessness of life.

Table S4. Estimated edge weights of the *"addiction"* profile

	dass02	dass04	dass07	dass09	dass15	dass19	dass20	dass03	dass05	dass10	dass13	dass16	dass17	dass21
dass02	0.00	0.15	0.20	0.13	0.00	0.03	0.20	0.00	0.04	0.00	0.00	0.02	0.05	0.00
dass04	0.15	0.00	0.04	0.05	0.00	0.07	0.18	0.09	0.00	0.00	0.00	0.05	0.04	0.00
dass07	0.20	0.04	0.00	0.00	0.15	0.05	0.04	0.15	0.01	0.00	0.00	0.10	0.00	0.00
dass09	0.13	0.05	0.00	0.00	0.11	0.06	0.02	0.12	0.00	0.04	0.24	0.05	0.00	0.00
dass15	0.00	0.00	0.15	0.11	0.00	0.00	0.21	0.20	0.00	0.01	0.23	0.00	0.00	0.09
dass19	0.03	0.07	0.05	0.06	0.00	0.00	0.05	0.00	0.07	0.00	0.00	0.12	0.15	0.01
dass20	0.20	0.18	0.04	0.02	0.21	0.05	0.00	0.00	0.04	0.00	0.00	0.02	0.12	0.00
dass03	0.00	0.09	0.15	0.12	0.20	0.00	0.00	0.00	0.17	0.22	0.11	0.00	0.00	0.03
dass05	0.04	0.00	0.01	0.00	0.00	0.07	0.04	0.17	0.00	0.09	0.00	0.08	0.14	0.24
dass10	0.00	0.00	0.00	0.04	0.01	0.00	0.00	0.22	0.09	0.00	0.13	0.06	0.00	0.23
dass13	0.00	0.00	0.00	0.24	0.23	0.00	0.00	0.11	0.00	0.13	0.00	0.20	0.12	0.18
dass16	0.02	0.05	0.10	0.05	0.00	0.12	0.02	0.00	0.08	0.06	0.20	0.00	0.01	0.00
dass17	0.05	0.04	0.00	0.00	0.00	0.15	0.12	0.00	0.14	0.00	0.12	0.01	0.00	0.22

dass21	0.00	0.00	0.00	0.00	0.09	0.01	0.00	0.03	0.24	0.23	0.18	0.00	0.22	0.00

Note: dass02: Dryness of mouth; dass03: No positive feeling; dass04: Breathing difficulty; dass05: Lack of motivation; dass07: Trembling; dass09: Worried about panicking; dass10: Nothing to live for; dass13: Downhearted and blue; dass15: Close to panic; dass16: Unable to get enthusiastic; dass17: Self-depreciation; dass19: Heart racing at rest; dass20: Unprovoked fear; dass21: Meaninglessness of life.

Figure S4. Estimation of edge weight difference by bootstrapped difference test

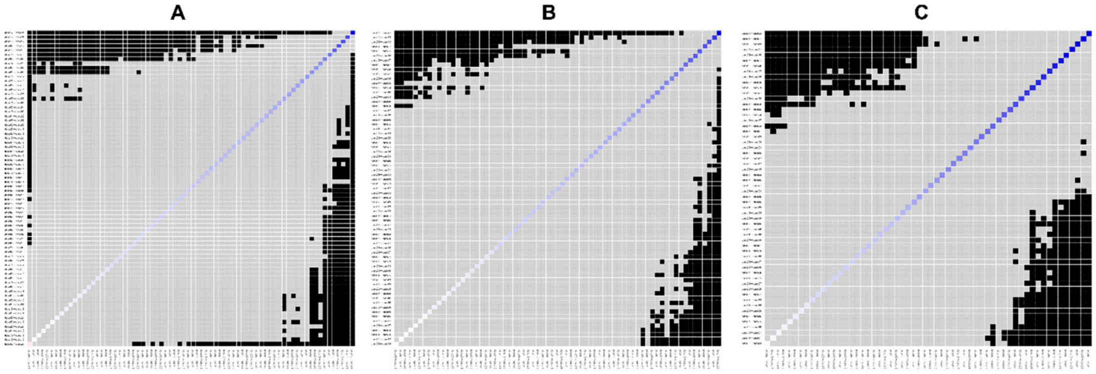

Note: A bootstrapped difference test was conducted between the edge weights in the network. The gray boxes indicate that edges are not significantly different from one another. The black boxes indicate significant differences. The blue boxes on the edge-weight plot represent positive correlations.

A: edge weight difference of *"regular"* profile; B: edge weight difference of *"risk"* profile; C: edge weight difference of *"addiction"* profile.

Figure S5. Estimation of node Expected Influence difference by bootstrapped difference test

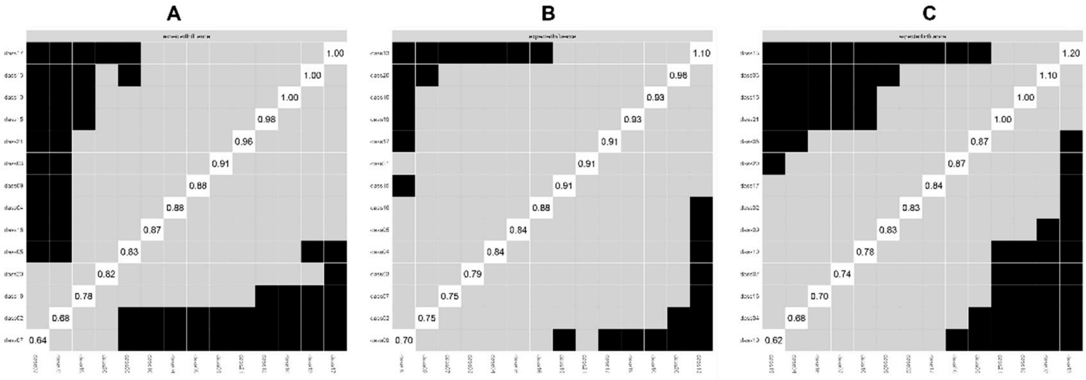

Note: Bootstrapped tests of node strength were conducted on 21nodes. The gray boxes indicate that edges are not significantly different from one another. The black boxes indicate significant differences. The white boxes represent the strength values for each node.

A: Expected Influence difference of *"regular"* profile; B: Expected Influence difference of *"risk"* profile; C: Expected Influence difference of *"addiction"* profile.

Figure S6. Estimation of node bridge Expected Influence difference by bootstrapped difference test

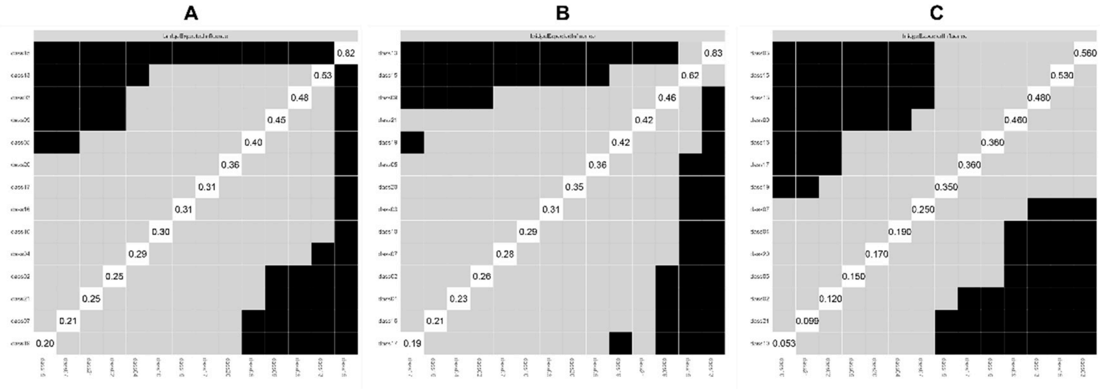

Note: Bootstrapped tests of node bridge Expected Influence were conducted on 21nodes. The gray boxes indicate that edges are not significantly different from one another. The black boxes indicate significant differences. The white boxes represent the strength values for each node.

A: bridge Expected Influence difference of *"regular"* profile; B: bridge Expected Influence difference of *"risk"* profile; C: bridge Expected Influence difference of *"addiction"* profile.

Figure S7. Comparison of network global strength

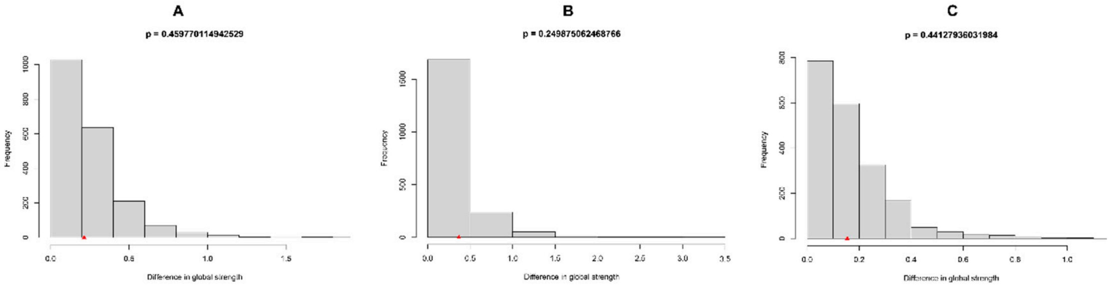

A: bootstrap value of the difference in *"regular"*- *"risk"* profile; B: bootstrap value of the difference in *"regular"* -*"addiction"* profile; C: bootstrap value of the difference in *"risk"*- *"addiction"* profile.

Figure S8. Comparison of network structure

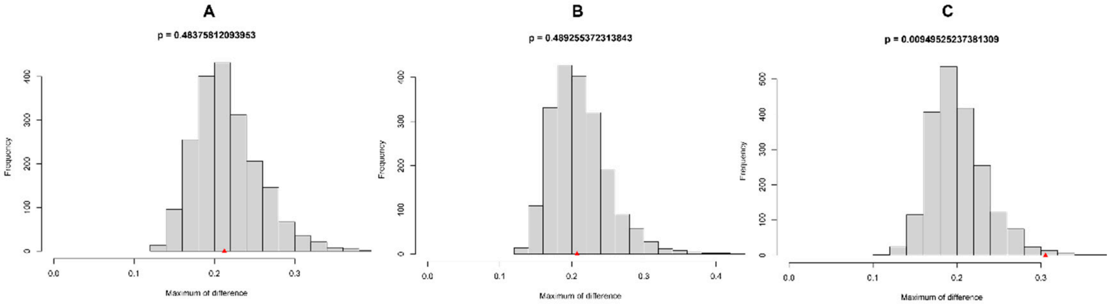

A: bootstrap value of the difference in *"regular"*-*"risk"* profile; B: bootstrap value of the difference in *"regular"* -*"addiction"* profile; C: bootstrap value of the difference in *"risk"*-*"addiction"* profile.
